# Potential of Soluble Decellularized Extracellular Matrix for Musculoskeletal Tissue Engineering – Comparison of Various Mesenchymal Tissues

**DOI:** 10.3389/fcell.2020.581972

**Published:** 2020-11-24

**Authors:** Hiroto Hanai, George Jacob, Shinichi Nakagawa, Rocky S. Tuan, Norimasa Nakamura, Kazunori Shimomura

**Affiliations:** ^1^Department of Orthopaedic Surgery, Osaka University Graduate School of Medicine, Suita, Japan; ^2^Department of Orthopaedics, Tejasvini Hospital, Mangalore, India; ^3^Center for Cellular and Molecular Engineering, Department of Orthopaedic Surgery, University of Pittsburgh, Pittsburgh, PA, United States; ^4^Institute for Tissue Engineering and Regenerative Medicine, The Chinese University of Hong Kong, Hong Kong, China; ^5^Institute for Medical Science in Sports, Osaka Health Science University, Osaka, Japan; ^6^Global Center for Medical Engineering and Informatics, Osaka University, Suita, Japan

**Keywords:** decellularized extracellular matrix, soluble factor, growth factor, mesenchymal tissue, tissue engineering

## Abstract

**Background:**

It is well studied that preparations of decellularized extracellular matrix (ECM) obtained from mesenchymal tissues can function as biological scaffolds to regenerate injured musculoskeletal tissues. Previously, we reported that soluble decellularized ECMs derived from meniscal tissue demonstrated excellent biocompatibility and produced meniscal regenerate with native meniscal anatomy and biochemical characteristics. We therefore hypothesized that decellularized mesenchymal tissue ECMs from various mesenchymal tissues should exhibit tissue-specific bioactivity. The purpose of this study was to test this hypothesis using porcine tissues, for potential applications in musculoskeletal tissue engineering.

**Methods:**

Nine types of porcine tissue, including cartilage, meniscus, ligament, tendon, muscle, synovium, fat pad, fat, and bone, were decellularized using established methods and solubilized. Although the current trend is to develop tissue specific decellularization protocols, we selected a simple standard protocol across all tissues using Triton X-100 and DNase/RNase after mincing to compare the outcome. The content of sulfated glycosaminoglycan (sGAG) and hydroxyproline were quantified to determine the biochemical composition of each tissue. Along with the concentration of several growth factors, known to be involved in tissue repair and/or maturation, including bFGF, IGF-1, VEGF, and TGF-β1. The effect of soluble ECMs on cell differentiation was explored by combining them with 3D collagen scaffold culturing human synovium derived mesenchymal stem cells (hSMSCs).

**Results:**

The decellularization of each tissue was performed and confirmed both histologically [hematoxylin and eosin (H&E) and 4’,6-diamidino-2-phenylindole (DAPI) staining] and on the basis of dsDNA quantification. The content of hydroxyproline of each tissue was relatively unchanged during the decellularization process when comparing the native and decellularized tissue. Cartilage and meniscus exhibited a significant decrease in sGAG content. The content of hydroxyproline in meniscus-derived ECM was the highest when compared with other tissues, while sGAG content in cartilage was the highest. Interestingly, a tissue-specific composition of most of the growth factors was measured in each soluble decellularized ECM and specific differentiation potential was particularly evident in cartilage, ligament and bone derived ECMs.

**Conclusion:**

In this study, soluble decellularized ECMs exhibited differences based on their tissue of origin and the present results are important going forward in the field of musculoskeletal regeneration therapy.

## Introduction

Musculoskeletal disorders are a prominent clinical problem in today’s population especially due to the increasing number of elderly people (Wolff et al., 2002). With age, musculoskeletal tissues degenerate significantly and result in bone fragility, loss of cartilage resilience, reduced ligament elasticity, loss of muscular strength, and fat redistribution, which deter the normal functioning of the bodied tissues (Freemont and Hoyland, 2007). Moreover, it is known that with age the body exhibits reduced healing potential and does not heal spontaneously. It is, therefore, a formidable clinical challenge to treat these disorders, with only a few currently available therapeutic strategies.

At present, tissue engineering and regenerative medicine have focused on extracellular matrices (ECMs) to function as a natural scaffold (Harrison et al., 2014). Such natural ECMs have been preferred as they contain many of the structural and bioactive components providing a natural microenvironment for seeded pluripotent cells used in tissue engineering (Yue, 2014). This overcomes many issues associated with synthetic scaffolds such as biocompatibility and degradability (Chan and Leong, 2008). However, natural tissues are a biological material, which raises concerns of immunologic reactions when transferred. Therefore, to mitigate immunogenic reactions the ECM cellular components must be removed by the process of decellularization (Gilpin and Yang, 2017). Decellularized ECM products of whole tissues have already been applied in clinical practice (Crapo et al., 2011).

While decellularized ECM products of whole tissues retain the ECMs basic morphology such as its biomechanical strength and high bioactive potency, it does have disadvantages such as size- and shape- mismatch and hampered cell infiltration due to its dense collagen structure (Ozasa et al., 2014; Schwarz et al., 2015). Past studies have focused on the soluble factors of decellularized ECMs derived from tendon, meniscus and cartilage and reported soluble factors of each tissue having tissue-specific and in some cases tissue region-specific bioactivity (Zhang et al., 2009; Rothrauff et al., 2017a,b; Shimomura et al., 2017). This indicates that each tissue or region constitutes different growth factors in varying amounts making them more or less ideal for application in the desired engineering of a specific tissue. Soluble factors extracted from each tissue have been thought to have the potential for application in various tissue regeneration therapies as they are effective as well as easy to handle in liquid form. We noted that past studies have only reported and compared two or three types of tissues at a time. Thus, we hypothesized that decellularized ECMs derived from different mesenchymal tissues could exhibit tissue-specific bioactivity. The purpose of this study is to compare the bioactivity of soluble decellularized ECMs obtained from various mesenchymal tissues and reveal the tissue-specific differences to investigate the content of sulfated glycosaminoglycan (sGAG), hydroxyproline and the concentration of several growth factors within each soluble factor of decellularized ECM. Finally, the effect of soluble ECMs on cell differentiation was explored by supplementing them into 3D collagen scaffolds culturing human synovium derived mesenchymal stem cells (hSMSCs) to confirm the bioactivity of each soluble ECM. In turn, these soluble ECMs may be utilized for future applications in musculoskeletal tissue engineering.

## Materials and Methods

### Tissue Decellularization

All experiments were conducted under the standard biosecurity and institutional safety procedures. Nine types of porcine tissues including cartilage, meniscus, ligament, tendon, muscle, synovium, fat pad, fat, and bone were harvested from the hindlimbs of 6–8-week-old pigs procured from a local slaughterhouse (Kasumi-syoji, Ibaraki, Japan) and stored at −20°C until use. After thawing the hindlimbs for preparation of the decellularized ECMs, each tissue fragment was harvested and then minced separately into small pieces. Bone tissue was collected from the anterior cortex of the midshaft of the tibia. For the preparation of decellularized ECMs a previously published protocol established for bovine meniscus and tendon, ECMs was utilized (Shimomura et al., 2017). Briefly, 4 g wet weight of minced tissues was agitated in 40 ml of phosphate-buffered saline (PBS; pH 7.4) containing with 1% Triton X-100 (Sigma-Aldrich, St. Louis, MO, United States) at 4°C for 3 days. Followed by three washes in PBS at 4°C for 30 min each, pieces of tissues were transferred to 40 ml of Hanks Buffered Salt Solution (HBSS, Thermo Fisher Scientific, Pittsburgh, PA, United States) supplemented with 200 U/ml DNase and 50 U/ml RNase (Worthington, Lakewood, NJ, United States) with continuous agitation at 37°C for 24 h. Finally, pieces of tissues were washed six times in PBS, as above. Decellularized tissues were stored at −20°C until the subsequent experiment. To confirm the complete decellularization, the absence of nuclei on both hematoxylin and eosin (H&E)-stained and 4’,6-diamidino-2-phenylindole (DAPI)-stained sections were observed and the content of double-stranded (ds) DNA per dry weight was calculated for each tissue, as described below.

### Histology of Native and Decellularized ECMs

Pieces of native and decellularized tissues were fixed in 10% phosphate buffered formalin, serially dehydrated, embedded in paraffin, followed by sectioned (3 μm thickness) with a microtome (REM-710, Yamato Koki, Saitama, Japan). Sample sections were rehydrated and stained with H&E or DAPI (Thermo Fisher Scientific). H&E-stained samples were examined with a slide scanner (Aperio CS2, Leica) while DAPI stained sections were imaged using an inverted fluorescent microscope (Eclipse 90i, Nikon Instruments, Tokyo, Japan) with excitation at 405 nm.

### dsDNA Quantification of Native and Decellularized ECMs

After overnight lyophilization to measure dry weight of ECMs, dried samples were digested overnight at 65°C in papain digestion reagent consisted of 0.2 M sodium phosphate buffer (Na_2_HPO_4_ – NaH_2_PO_4_, pH 6.4) with 0.1 M sodium acetate, 0.01 M EDTA, disodium salt, 5 mM cysteine HCl, and 0.5 v/v% papain (crystallized suspension, Sigma-Aldrich). The dsDNA content in the supernatant of the papain digested samples was measured, in duplicate, with Quant-iT PicoGreen dsDNA Assay Kit (Thermo Fisher Scientific) using a fluorescence microplate reader (excitation 485 nm, emission 535 nm, SH-9000Lab, Hitachi High-Tech, Tokyo, Japan) according to the manufacturer’s instructions.

### Biochemical Composition of Native and Decellularized ECMs

To quantify sGAG content the papain digested samples (see above) were treated with a Blyscan Glycosaminoglycan Assay Kit (Biocolor, Carrickfergus, United Kingdom) according to the manufacturer’s instructions. Dilutions of the provided bovine tracheal chondroitin 4-sulfate were used to generate a standard curve and the absorbance of each sample was measured, in duplicate, on a spectrophotometer at 656 nm (Multiscan Go, Thermo Fisher Scientific). The hydroxyproline content was determined to be the amount of total collagen as hydroxyproline is present almost exclusively in collagen. This was determined using a modified hydroxyproline assay (Cissell et al., 2017). Briefly, 200 μl of each papain digested sample was hydrolyzed with an equal volume of 4 N NaOH at 95°C overnight, followed by cooling to room temperature. It was then neutralized with 200 μl of 4 N HCl. Subsequently, 100 μl of the neutralized solution was combined with 200 μl chloramine-T solution containing 0.05 M chloramine-T (Nacalai tesque, Kyoto, Japan) in 74% v/v H_2_O, 26% v/v 2-propanol, 0.629 M NaOH, 0.140 M citric acid (anhydrous), 0.453 M sodium acetate (trihydrate), and 0.112 M acetic acid and allowed to stand at room temperature for 20 min. The solution was then combined with 200 μl of Ehrlich’s solution consisting of 1M p-dimethylaminobenzaldehyde (DMAB, Nacalai tesque) in 30% v/v HCl and 70% v/v 2-propanol and incubated at 65°C for 20 min. 200 μl of each sample was transferred to a clear 96-well plate, in duplicate, and absorbance at 550 nm was read. Serial dilutions of L-hydroxyproline (Wako, Osaka, Japan) was prepared as a standard curve.

### Solubilization of Decellularized ECM

A water-soluble fraction of decellularized ECM was extracted by urea solution, as previously described (Shimomura et al., 2017). Briefly, 4 g of wet decellularized ECM was powdered using a Freezer/Mill 6770 (SPEX Sample Prep, Metuchen, NJ, United States) and then agitated in 40 ml of 3 M urea (Sigma-Aldrich) in water at 4°C for 3 days. The suspension was then centrifuged for 20 min at 1,500 g and the supernatant was then transferred to a benzoylated dialysis tube (pore size; 2,000 MWCO, Sigma-Aldrich) and dialyzed against ddH2O for 2 days at 4°C. Water changes were done every 12 h. After removal of urea, the water-soluble ECM was transferred into centrifugal filter tubes (pore size; 3,000 MWCO, Merk Millipore, Billerica, MA, United States) and spin-concentrated approximately 10-fold at 4,000 g for 30 min. Protein concentration was determined by performing a BCA assay (Thermo Fisher Scientific) according to the manufacturer’s instructions and the soluble ECMs were stored at −80°C until further use.

### SDS-PAGE and Gel Staining

Each urea-extracted sample was suspended in RIPA buffer (Thermo Fisher Scientific). One μg total protein was mixed with a loading buffer (NuPAGE, Thermo Fisher Scientific) and dithiothreitol (DTT) used as a reducing agent and heated for 10 min at 70°C. The protein was loaded into a pre-cast 12-well 4–12% Bis-Tris gel (Thermo Fisher Scientific) and separated by electrophoresis in MOPS running buffer for 35 min at constant 200 V. The gel was stained with Silver Stain MS Kit (Wako) according to the manufacturer’s instructions. The stained gel was photographed using a CCD camera gel imaging system (ChemiDoc Touch, Bio-Rad, Hercules, CA, United States).

### Growth Factor Analysis of Soluble ECM

The amounts of basic fibroblast growth factor (bFGF), insulin-like growth factor-1 (IGF-1), vascular endothelial growth factor (VEGF), transforming growth factor-β1 (TGF-β1), bone morphogenic protein-2 (BMP-2) and growth differentitaion factor 7 (GDF-7) in the extracted solution were measured by enzyme-linked immunosorbent assay (ELISA) kits for human purchased from R&D Systems (Minneapolis, MN, United States) for bFGF, IGF-1, VEGF, TGF-β1,and BMP-2, Biocompare (South San Francisco, CA, United States) for GDF-7 according to the manufacturer’s instructions. The concentration of growth factor was calculated in 500 μg/ml soluble ECM preparations.

### Cell Isolation and Culture

Human synovium derived mesenchymal stem cells were isolated and expanded as previously described (Ando et al., 2007; Koizumi et al., 2016). Synovium was obtained from an 18-year-old male donor who underwent arthroscopic surgery for an anterior crucial ligament reconstruction in accordance with the approvals of the institutional committee for medical ethics. hSMSCs were cultured in growth medium containing high-glucose Dulbecco’s Modified Eagle’s Medium (DMEM, Nacalai tesque), supplemented with 10% fetal bovine serum (FBS, Sigma-Aldrich) and 1% antibiotic-antimycotic solution (Sigma-Aldrich) at 37°C with humidified 5% CO_2_. At 80% confluence, cells were detached with 0.25% trypsin in 1 mM EDTA (Thermo Fisher Scientific) and passaged. All experiments were performed with passage 4 hSMSCs.

### 3D Culture With Collagen Gel

The hSMSCs were trypsinized from cell culture dishes and embedded in a collagen gel. Eight volumes of Cellmatrix type I-A (Nitta Gelatin, Osaka, Japan) were mixed with both one of 10 × MEM and the reconstitution buffer according to the manufacturer’s instructions. hSMSCs were suspended in the collagen mixture at a density of 1.0 × 10^7^ cells/ml of gel, containing a final concentration of either 100 mg/mL of each soluble ECM or the equivalent volume of PBS as a control. Then, 15 μl of collagen mixture containing cells and soluble ECMs were added into 1.5 ml conical tubes. After the gel forming by incubation, 0.5 ml of reduced-serum medium (DMEM with 2% FBS and 1% antibiotics) was added to each tube and changed every 3 days.

### Gene Expression Analysis

On culture days 3 and 7 for 3D culture, RNA isolation was preceded by homogenization of samples in Trizol (Thermo Fisher Scientific) and then extracted using a Direct-zol RNA Microprep Kit (Zymo Research, Irvine, CA, United States) according to the manufacturer’s protocol. Total RNA was reverse transcribed into complementary DNA through use of the ReverTra Ace qPCR RT Master Mix with gDNA Remover (Toyobo, Osaka, Japan). Quantitative PCR (qPCR) was performed using SYBR Green Master Mix in the Step One Plus real-time PCR system (Thermo Fisher Scientific). Data were normalized to GAPDH and relative expression of each target was calculated according to the 2^–Δ^ ^Δ^ ^*Ct*^ formula. Ten kinds of genes were selected for investigation, including SOX9, ACAN, SCX, TNC, Desmin, PPARG, RUNX2, COL1A1, COL2A1, and COL3A1. The targets and sequences of primers are shown in Table 1.

**TABLE 1:** Target gene primer sequences for quantitative PCR (qPCR).

Gene	Primer Sequence (5′-3′)		Product Size (bp)
*GAPDH*	Forward	CAAGGCTGAGAACGGGAAGC	194
	Reverse	AGGGGGCAGAGATGATGACC	
*SOX9*	Forward	CTGAGCAGCGACGTCATCTC	72
	Reverse	GTTGGGCGGCAGGTACTG	
*ACAN*	Forward	AGGCAGCGTGATCCTTACC	137
	Reverse	GGCCTCTCCAGTCTCATTCTC	
*SCX*	Forward	TGCGAATCGCTGTCTTTC	91
	Reverse	GAGAACACCCAGCCCAAA	
*TNC*	Forward	TTCACTGGAGCTGACTGTGG	223
	Reverse	TAGGGCAGCTCATGTCACTG	
*Desmin*	Forward	CTGAGCAAAGGGGTTCTGAG	109
	Reverse	ACTTCATGCTGCTGCTGTGT	
*PPARG*	Forward	GGCTTCATGACAAGGGAGTTTC	74
	Reverse	AACTCAAACTTGGGCTCCATAAAG	
*RUNX2*	Forward	CAACCACAGAACCACAAGTGCG	196
	Reverse	TGTTTGATGCCATAGTCCCTCC	
*COL1A1*	Forward	TAAAGGGTCACCGTGGCT	355
	Reverse	CGAACCACATTGGCATCA	
*COL2A1*	Forward	CGTCCAGATGACCTTCCTACG	122
	Reverse	TGAGCAGGGCCTTCTTGAG	
*COL3A1*	Forward	CAGCGGTTCTCCAGGCAAGG	179
	Reverse	CTCCAGTGATCCCAGCAATCC	

### Statistical Analysis

All quantitative data were reported as mean ± standard deviation (SD). Student’s t-test for DNA content or one-way analysis of variance (one-way ANOVA) for growth factor concentration and relative gene expression levels or two-way ANOVA for hydroxyproline and sGAG content followed by Tukey’s *post hoc* test or Dunnett’s test were performed and analyzed with JMP pro 14.0 (SAS Institute, Cary, NC, United States) and significance was set as *p* < 0.05.

## Results

### Decellularization of ECMs

**FIGURE 1:**
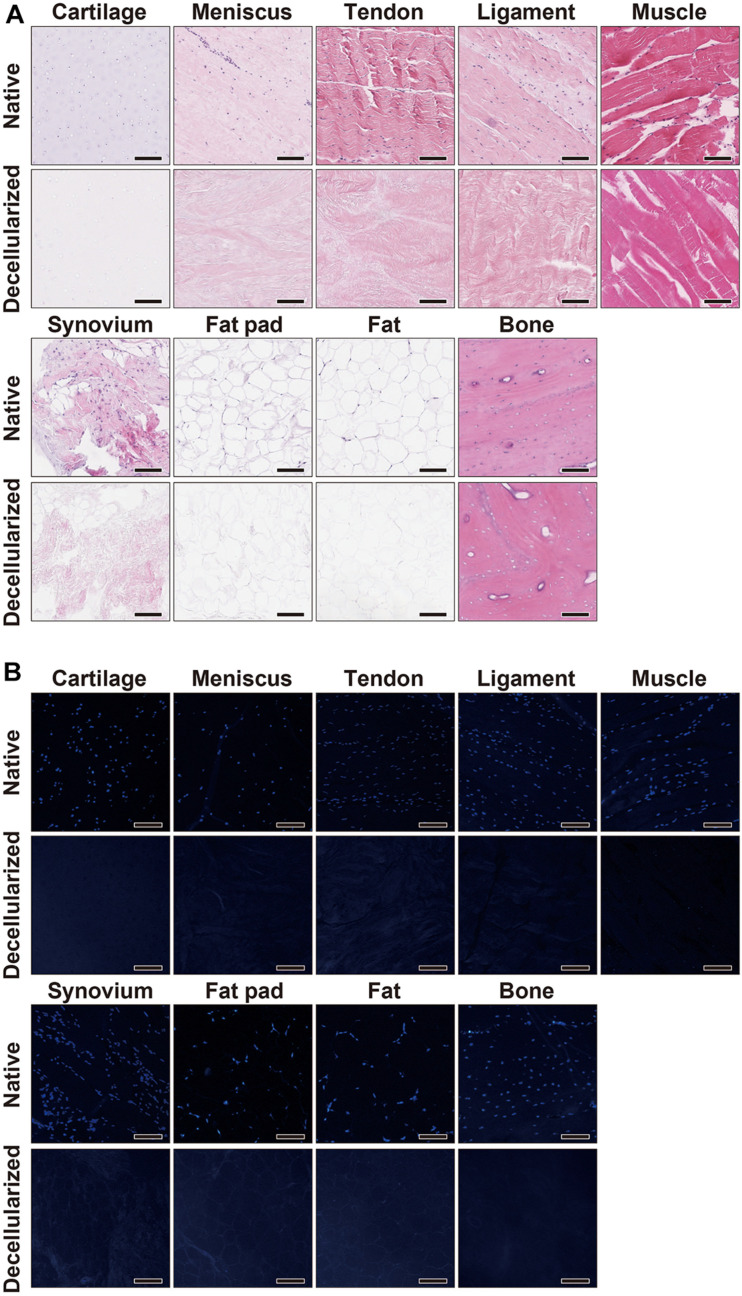
Histological characterization of native and decellularized tissue. **(A)** H&E and **(B)** DAPI staining of each native and decellularized tissue; Scale bar = 100 μm. H&E, hematoxylin and eosin; DAPI, 4’,6-diamidino-2- phenylindole.

**FIGURE 2:**
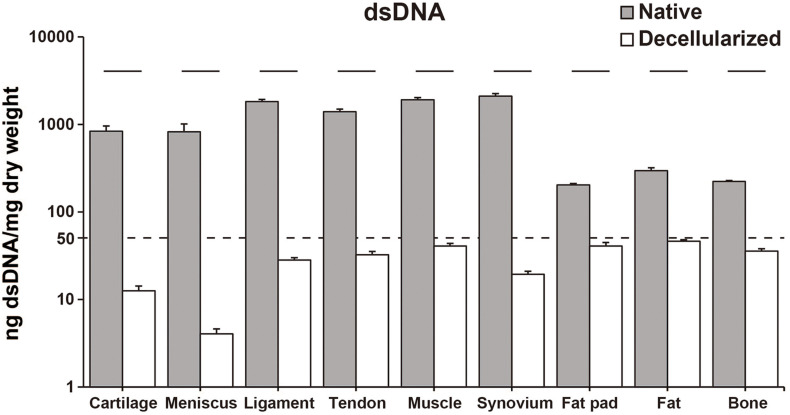
dsDNA content of native and decellularized tissue. *Y*-axis was indicated with logarithmic scale; dotted line at 50 ng/mg is established threshold for sufficient decellularization. *n* = 5 per condition. Lines over bars indicate significant difference between native and decellularized tissue of each region, *p* < 0.001. dsDNA, double stranded DNA.

The decellularization protocol with Triton X-100 treatment and nuclease enzymes reduced cellular content from every tissue, and this was verified by histology with H&E staining (Figure 1A). When compared to native tissues the morphology of the decellularized tissue was reasonably preserved in each tissue. Furthermore, DAPI staining also confirmed the absence of cell nuclei (Figure 1B). The DNA content of each decellularized tissue was significantly reduced compared with that of its original tissue (e.g., native vs. decellularized meniscus: 836.9 ± 190.3 ng/mg vs. 12.6 ± 1.7 ng/mg, *p* < 0.001; native vs. decellularized fat pad: 204.1 ± 7.9 ng/mg vs. 40.9 ± 3.8 ng/mg, *p* < 0.001; a similar trend was observed in the rest of the tissues tested; Figure 2). This confirmed the successful decellularization of the tissues.

### Hydroxyproline and sGAG Content

**FIGURE 3:**
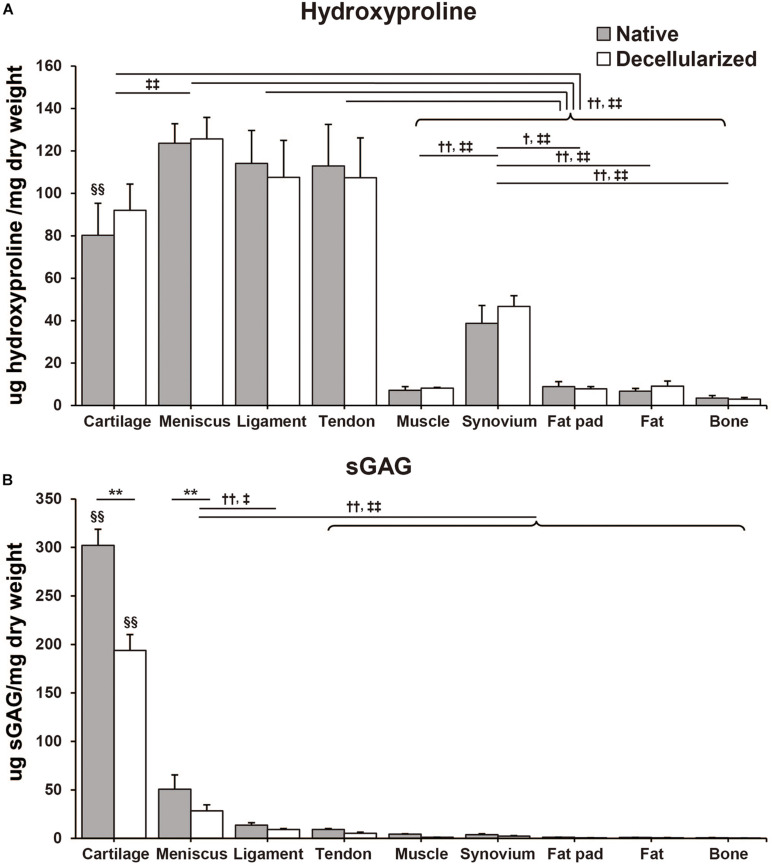
Biochemical composition of native and decellularized tissue. **(A)** Total hydroxyproline and **(B)** total sulfated glycosaminoglycan (sGAG) content in native and decellularized tissue. *n* = 5 per condition. **p < 0.01, significant difference between native and decellularized tissue for each step. ^†^*p* < 0.05, ^†⁣†^*p* < 0.01, significant difference between regions in a given native tissue. ^‡^*p* < 0.05, ^‡⁣‡^*p* < 0.01, significant difference between regions in a given decellularized tissue. ^§§^ *p* < 0.01, significant difference from any other tissues in the same step.

Hydroxyproline content was quantified by determining the total amount of collagen in the native and decellularized tissue. Per dry weight hydroxyproline content was maintained during the decellularization process and there were no significant differences between native and decellularized tissue (Figure 3A). The content of hydroxyproline in the meniscus-derived ECM was the highest (native: 123.6 ± 15.1 μg/mg; decellularized: 125.7 ± 12.4 μg/mg) when compared to other tissues closely followed by that of ligament and tendon tissue (native: 114.1 ± 15.5 μg/mg, *p* = 0.99 and 113.0 ± 19.5 μg/mg, *p* = 0.99; decellularized: 107.6 ± 10.4 μg/mg, *p* = 0.57 and 107.3 ± 18.9 μg/mg, *p* = 0.55, respectively). The difference between the hydroxyproline content of meniscus, tendon and ligament tissue was not significant. When compared to muscle, synovium, fat pad, fat, and bone the hydroxyproline content was significantly higher in meniscus, ligament, tendon, and cartilage tissue (*p* < 0.01). When studying the sGAG content, cartilage tissue demonstrated significantly higher sGAG content than any of the other tissues followed by meniscus and then ligament tissue (Cartilage; native: 302.1 ± 16.6 μg/mg, *p* < 0.01, decellularized: 193.9 ± 16.4 μg/mg, *p* < 0.01. Meniscus; native: 50.6 ± 14.9 μg/mg, *p* < 0.01; decellularized: 28.5 ± 6.2 μg/mg, *p* < 0.01 except for when compared to decellularized ligament, *p* = 0.013; Figure 3B). In the decellularization process, cartilage- and meniscus-derived decellularized ECMs significantly lost their sGAG content when compared to that of their native tissue (*p* < 0.01). The remaining seven types of tissues also showed 32–72% loss of the sGAG content but the difference between their native and decellularized state was no significant.

### Total Protein and Growth Factor Distribution

**FIGURE 4:**
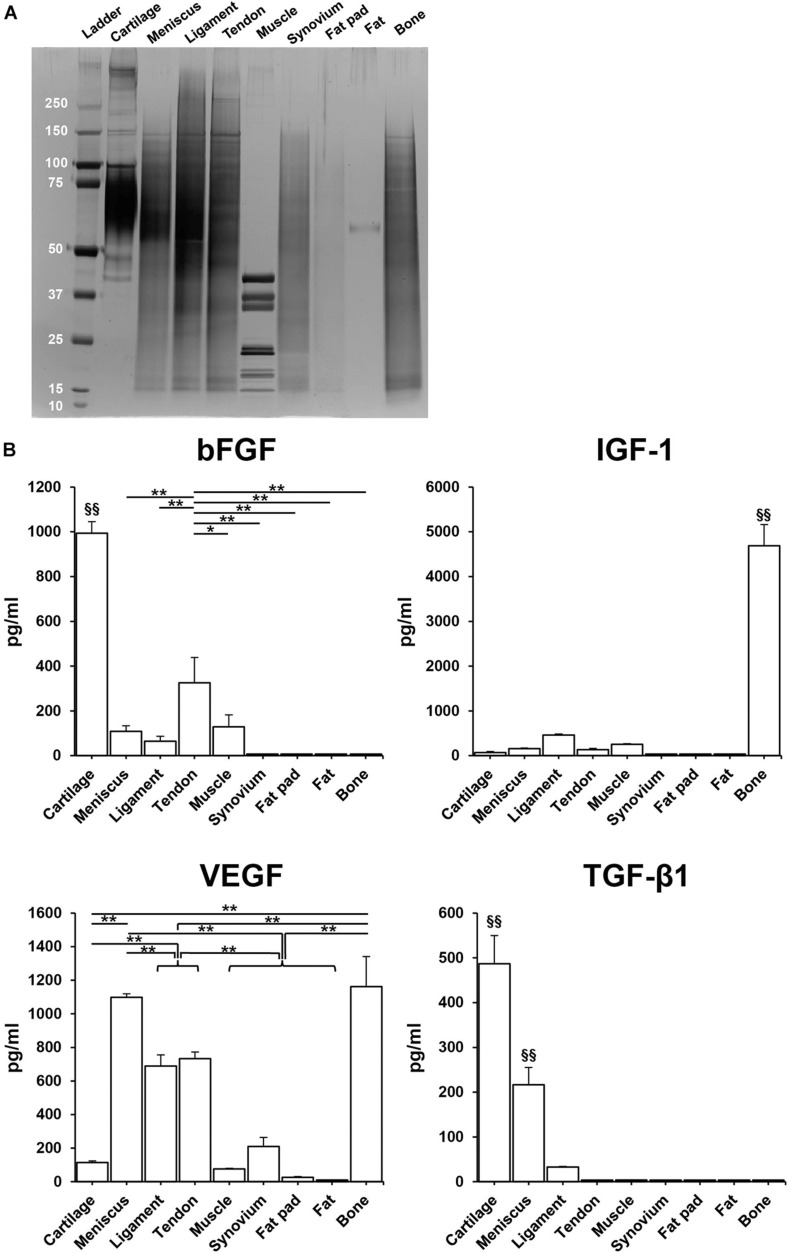
Total protein and human growth factor analysis of soluble decellularized extracellular matrix (ECM) preparations. **(A)** SDS-PAGE analysis of each urea-extracted tissue. **(B)** Growth factor concentrations (pg/ml) in 500 μg/ml soluble ECM preparations. *n* = 3 per condition. *p < 0.05, **p < 0.01, significant difference from each preparation. ^§§^ *p* < 0.01, significant difference from any other preparations. bFGF, basic fibroblast growth factor; IGF-1, insulin-like growth factor-1; VEGF, vascular endothelial growth factor; TGF-β1, transforming growth factor-β1.

SDS-PAGE showed that the urea-extracted protein distribution of each tissue was different, but most samples were enriched for low to moderate molecular weight proteins. Tissues derived from fat pad and fat exhibited less content of protein (Figure 4A). ELISA analysis confirmed the presence of various growth factors in the different solubilized decellularized ECMs (Figure 4B). The amount of bFGF in cartilage was significantly higher than the other tissues (993.5 ± 51.5 pg/ml, *p* < 0.01), followed by tendon and then muscle. In bone tissue, IGF-1 content was found to be significantly higher than the other studied tissues (4688.1 ± 51.5 pg/ml, *p* < 0.01). VEGF presence in bone and meniscus (1162.0 ± 179.6 pg/ml, 1098.6 ± 20.8 pg/ml, respectively) was significantly greater than the other seven studied tissues, followed by tendon and ligament. The concentration of TGF-β1 in cartilage (498.6 ± 62.8 pg/ml) was significantly higher when compared to other tissues-derived soluble factors, followed by meniscus tissue. Both GDF-7and BMP-2 were not detected in any of the sample preparations.

### Gene Expression Profiles During 3D Culturing

**FIGURE 5:**
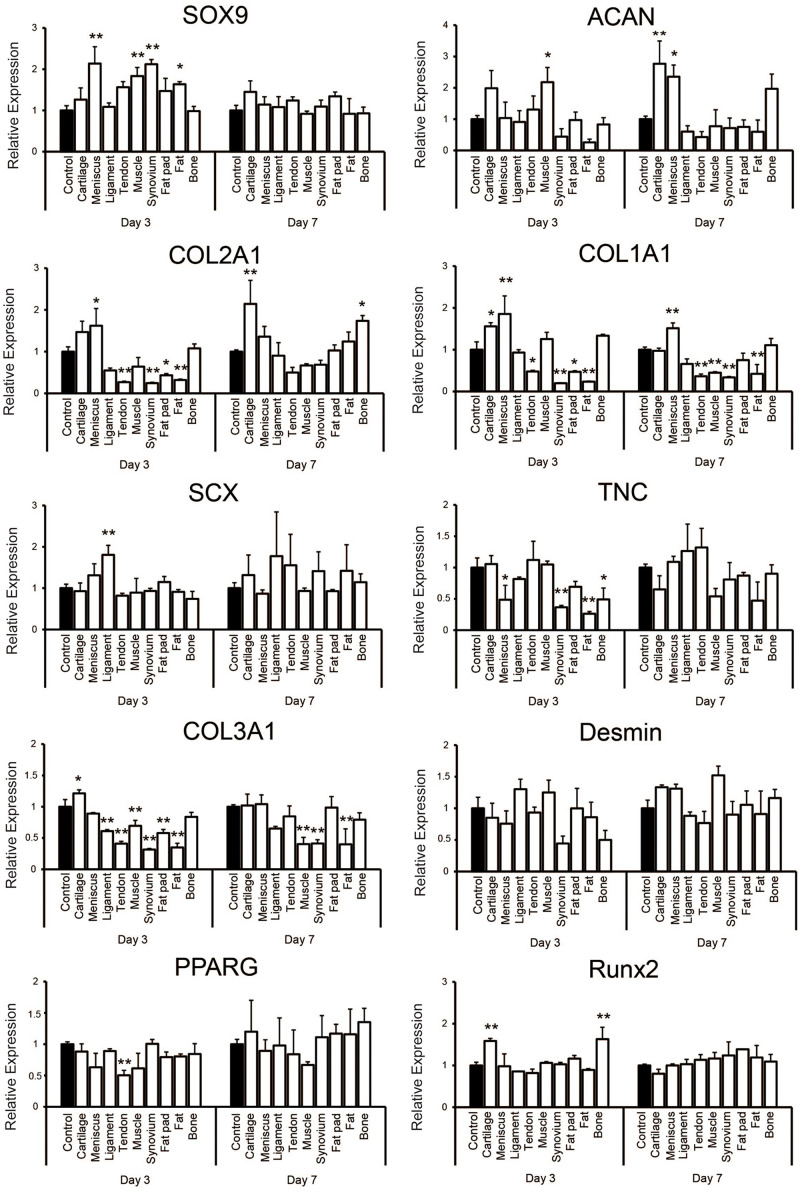
Gene expression analysis of human synovium derived mesenchymal stem cells (hSMSCs) seeded in soluble decellularized extracellular matrix (ECM)-supplemented scaffolds (3D collagen + ECM) on days 3 and 7. Each gene expression level was normalized to GAPDH and showed as relative expression levels compared to the control group of the respective day. *n* = 3 per condition. *p < 0.05, **p < 0.01, significant difference compared to the control group.

Quantitative PCR showed that supplementation with each tissue-derived soluble ECM varied the relative level of some gene expressions on culture days 3 and 7 when compared to the control group (Figure 5). The expression of SOX9 was significantly higher on day 3 of culture in tissues including meniscus, synovium, muscle and fat. It was upregulated slightly in the cartilage tissue on day 7 (1.45 ± 0.26 -fold change compared to the control), but there was no significant difference in any groups when compared to the control group. ACAN was significantly higher in the cartilage and meniscus group on day 7 (*p* < 0.01 and *p* < 0.05, respectively). The expression level of COL2A1 was significantly higher in the cartilage group when compared to the control on day 7 (*p* < 0.01). On the other hand, COL1A1 was significantly upregulated in the meniscus tissue on days 3 and 7 (*p* < 0.01). We also noted SCX to be significantly higher in the ligament group on day 3 (*p* < 0.01) and was further upregulated in the ligament and tendon group compared to the control (1.78 ± 1.07 or 1.55 ± 0.75 fold change, respectively) on day 7, but not significantly. TNC was not significantly higher in the ligament and tendon group when compared to the control on days 3 and 7. However, COL3A1 expression was significantly lower in the ligament and tendon group when compared to the control on day 3 (*p* < 0.01) and still lower on day 7, although without significance. Desmin was upregulated in the muscle group on day 7 (1.52 ± 0.14 -fold change compared to the control group) with the tendency of difference (*p* = 0.056). There was no upregulation of expression of PPARG in any group compared to the control. RUNX2 was significantly higher in the cartilage and bone group on day 3 (*p* < 0.01). Taken together, these findings demonstrated that hSMSCs exposed to each tissue-derived soluble ECM in 3D culture exhibited varying gene expression levels that suggested differentiation toward a certain tissue based on the ECM tissue source used may be possible.

## Discussion

Our study applied one decellularization protocol to nine types of porcine tissue and compared the differences of biochemical composition in each decellularized tissue and the distributions of several growth factors and bioactivities in each soluble fragment. Our findings revealed that decellularization was successful with minimal disruption to the original tissue morphology. Hydroxyproline content was retained in all of the tissues post the decellularization protocol with some tissue specific variations noted. sGAG content was reduced post decellularization, notably in cartilage and meniscus which contained the highest amount in their native states. Moreover, several growth factors important for cell proliferation, migration and differentiation such as bFGF, IGF-1, VEGF, and TGF-β1 were detected in the tissues in varying amounts and each tissue-derived soluble ECM behaved with dissimilar bioactivity. Further studies will be needed, but soluble decellularized ECMs may be feasible to repair and regenerate injured musculoskeletal tissues and matching the decellularized tissue ECM to the desired tissue regenerate may allow for a more effective tissue engineering method. Thus, our results are important going forward in the field of musculoskeletal regeneration therapy to construct effective tissue specific ECMs.

Tissue ECMs make up the non-cellular components of all tissues and have been shown to provide important signaling for cell migration, proliferation as well as providing an essential physical 3-D scaffolding for the cells. Together these features contribute to the biochemical and biomechanical roles a tissue requires to undergo morphogenesis, differentiation and to maintain a homeostatic environment (Frantz et al., 2010; Vorotnikova et al., 2010; Crapo et al., 2011; Yue, 2014). Due to these advantages, ECMs have been regarded as an ideal scaffold material for tissue engineering especially when engineering an identical tissue (Valentin et al., 2006; Badylak et al., 2009). The native tissues possess genetic cellular material which can elicit harmful immunologic reactions therefore when used in a clinical setting all cellular material must be removed by the process of decellularization (Brown et al., 2009; Nagata et al., 2010; Zhang et al., 2010). To eliminate the cellular components and reduce immune reactions without extensively damaging the ECM, numerous decellularization protocols have been described. The optimal technique for decellularization depends on the structure and tissue cellularity (Petersen et al., 2010; Lehr et al., 2011; Yang et al., 2013). Decellularized ECM products of whole tissues have been already applied in clinical practice (Crapo et al., 2011).

There are still concerns about that the dense collagenous architecture of ECM acting as a barrier for cell infiltration, with cells often localized only to the tissue surface (Ozasa et al., 2014; Schwarz et al., 2015). Moreover, the use of whole decellularized tissues as grafts is limited because of size and shape as well as the immunogenicity elicited between donors and recipients. To overcome these problems while retaining the tissue-specific bioactivity in the ECM, decellularized tissues have been processed into powder form (Almeida et al., 2016; Beck et al., 2016; Rowland et al., 2016) or solubilized with enzymatic (like pepsin) or chaotropic agents (like urea), resulting in easy-to-handle solutions (Kwon et al., 2013; Farnebo et al., 2014; Kim et al., 2014; Pati et al., 2014). Urea extracted ECMs are superior to pepsin digested ECMs as they retain several growth factors. These growth factors have been noted to promote tissue-specific cell phenotypes and differentiation, indicating that urea extracted decellularized ECMs possess tissue-specific growth factors (Zhang et al., 2009; Lin et al., 2012; Yang et al., 2013; Rothrauff et al., 2017b). Past studies have only reported and compared two or three types of tissues. Zhang et al. (2009) reported that urea-extracted fractions of decellularized ECM from the skin, skeletal muscle and liver tissues revealed significant differences in adhesion properties, growth rates and promoting tissue-specific differentiation. Rothrauff et al. (2017b) compared cartilage and tendon growth factors and reported promotion of tissue-specific differentiation across multiple cultures and also reported the distributions of various growth factors. Lee et al. (2019) confirmed the decellularization of seven tissues including organs such as liver and heart. They fabricated uniform sized tissue microbeads using them ECM and reported three kinds of tissue-specific microbeads derived from liver, heart and muscle (Lee et al., 2019). These significantly enhanced the viability, lineage specific maturation, and functionality of each type of reprogrammed cell, when compared to conventional microbeads from collagen components.

In our present study, the tissue-specific differences of hydroxyproline, sGAG, growth factors and bioactivities in nine types of decellularized porcine mesenchymal tissues including cartilage, meniscus, ligament, tendon, muscle, synovium, fat pad, fat, and bone were investigated. Minimal criteria of successful decellularization was reported as < 50 ng dsDNA/mg ECM dry weight, < 200 bp DNA fragment length and the lack of visible nuclear material in tissue sections stained with DAPI or H&E (Crapo et al., 2011). The decellularization technique in our present study was previously reported successful for bovine tendon, meniscus and cartilage tissues with significant reduction in DNA content and absence of cellular nuclei by DAPI staining (Yang et al., 2013; Rothrauff et al., 2017a,b; Shimomura et al., 2017). Other tissues were decellularized with a similar protocol based on Triton-X, such as for ligament combined with nucleases (Vavken et al., 2009) or for muscle after 1 h exposure to trypsin/EDTA but without nucleases (Stern et al., 2009). Reisbig et al. (2016) pointed out that decellularized synovium incubated with 1% Triton X-100 followed by DNase had low DNA content and short DNA fragments, but the synovial villous architecture was destroyed and therefore suggested using peracetic acid was better methods. Adipose tissue was decellularized by Triton-X combined with nucleases, but the result was not sufficient reduction of cells or cell fragments (Sano et al., 2014). While Triton-X was exposed for only 16 h in their protocol, the present protocol was for 72 h, which may cause to reduce cell fragments more. Bone tissue were decellularized Triton-X after freeze and thermal shock and followed by incubation with ethanol and then the reduction in DNA content was higher than 90% compared to that of native bone (Gardin et al., 2015). On the other hand, considering that the reduction rate of dsDNA in fat pad, fat or bone tissue is around 80% and the dry weight of them included fat or mineral component, the present method might be not so effective for all tissues. Triton-X could be one of the most standard process for decellularization, but residual DNA may remain present in the tissue (Yang et al., 2017). Therefore, enzymatic treatments are used in the final decellularization step to reduce any residual DNA content. At the same time it is already known that it is not the best process for most tissues and the most effective agents for decellularization of each tissue will depend upon many factors, including the tissue’s cellularity, density, lipid content, and thickness (Crapo et al., 2011). For example fatty, amorphous organs and tissues such as adipose tissue typically require the addition of lipid solvents such as alcohols (Flynn, 2010). The optimized tissue-specific decellularized methods which preserve tissue-specific key ECM components for orthopedic tissue engineering can be found in recent comprehensive reviews (Cheng et al., 2014; Mendibil et al., 2020). Considering the effectiveness of decellularization or preservation of the components of each tissue-derived ECM, it might be better to select the appropriate protocol depend on tissue, but a single protocol same as our past study was chosen because we would like to expand our past procedure to the present tissues and compare them.

We noted hydroxyproline content was retained in all of the tissues after the decellularization protocol indicating good retention of collagen content. On the other hand, native meniscus and cartilage tissue had a high amount of sGAG which was significantly reduced after decellularization. The remaining tissues also demonstrated 32–72% reduction in sGAG, however, they did not have a high sGAG content to begin with. The variations in native collagen content can be explained by the functional roles of the tissue and the type and magnitude of stresses applied on them. Eleswarapu et al. (2011) reported hyaline tissue to have high collagen and sGAG content while fibrous tissue such as ligaments and tendons to have high collagen, and low sGAG content. Hyaline tissue experiences a balance of compressive and tensile forces while fibrous tissues mainly experience tensile stresses on locomotion. Another study concluded that baseline muscle collagen content was much lower when compared to the collagen content of dense connective tissues (tendons and aponeuroses) in murine native legs (Binder-Markey et al., 2020). We also noted synovial tissue to possess a reasonable collagen content. In spite of the joint synovial membrane lacking a continuous basement membrane the cells on the surface of the synovial membrane are supported by a loose fibrillary network containing a mixture of fibers derived from Type I and III collagen molecules (Gay et al., 1980). With regard to bone tissue, it’s organic matrix contains type I collagen, which constitutes 85–95% of the matrix (Rogers et al., 1952). In our study papain was used as a digestion buffer for each decellularized ECM. It was likely that sGAG and collagen contents could have been underestimated due to the use of a mild digestion which was expected to high amount of insoluble material.

We also studied growth factor contents within the various mesenchymal tissues and key growth factors for cell proliferation, migration and differentiation were detected in varying amounts in each different tissue. bFGF, also known as FGF-2 plays various roles in fibroblast proliferation, migration, angiogenesis but also promotes differentiation (Fujita et al., 2005; Yun et al., 2010). The possible effects of bFGF on myogenesis, adipogenesis, tenogenesis, and osteogenesis along with regeneration of these tissues has been reported (Webb et al., 1997; Kawaguchi et al., 1998; Doukas et al., 2002; Jeong et al., 2010). bFGF is produced by chondrocytes and stored within the ECM (Ellman et al., 2013). It aids in collagen and glycosaminoglycan synthesis and helps maintain stem cells in a undifferentiated state (Shida et al., 2001). Another important growth factor is insulin-like growth factor-I (IGF-I) which plays a crucial role in muscle and bone regeneration (Adams and McCue, 1998; Trippel, 1998; Titan et al., 2019). IGF-I mediates to be largely proliferation and differentiation of satellite cells as well as recruitment of bone marrow stem cells (Musaro et al., 2004; Provenzano et al., 2007). Furthermore, IGF-I is involved in numerous physiologic processes and promotes healing in tissues such as cartilage, skin and tendon (Dahlgren et al., 2002; Zhang et al., 2017). Though literature has determined the potential roles of various growth factors, their tissue-specific distribution is not certain. In our study we found the amount of bFGF to be increased in cartilage tissue, followed by tendon and muscle. However, bFGF content in fat, fat pad, synovium and bone were negligible. IGF-1 content was very high in bone tissue even without demineralization. VEGF is an important angiogenic factor which increases vascular permeability and vascular endothelial cell proliferation (Ferrara, 1999; Turnbull et al., 2018). We expected a high concentration of VEGF in the vascular-rich tissues such as muscle and bone, however, interestingly we noted a high level in the meniscus tissue, while that in muscle was low. The meniscus has been described to have heterogeneous structure, and possesses a vascularity only in its middle and outer zones (Mauck and Burdick, 2015; Jacob et al., 2019). Porcine menisci has also been described to have increased VEGF content from the inner to the outer zone, explaining why the meniscus tissue expressed a high VEGF concentration (Di Giancamillo et al., 2017). Moreover, as the concentration of growth factor was calculated to per unit protein not per tissue weight in this study, the distributions in soluble factor of each tissue may be different from that we have expected. The final growth factor that we studied was TGF-β1 which is member of a family of numerous ligands essential for development and cell homeostasis (Mao and Mooney, 2015; Kwak and Lee, 2019). TGF-β1 has been frequently employed in tissue engineering to support cell growth, adhesion and proliferation making it essential component for successful regeneration of tissue (Kwak and Lee, 2019). TGF-β1 is particularly abundant in cartilage tissue and helps in promoting matrix synthesis in articular chondrocytes without which the chondrocyte phenotype resemble that of osteoarthritic tissue (Blaney Davidson et al., 2007; Finnson et al., 2012). This is aided by the presence of decorin, biglycan, and chondroitin sulfate which keeps TGF-β1 within the pericellular matrix (Lindahl et al., 2015). Our results also indicate and confirm increased presence of TGF-β1 in cartilage tissue followed by meniscus which does possess cells with chondrocyte-like-morphology (Wilson et al., 2009). Other factors such as GDF-7 and BMP-2 were not found in the tissues though we expected to find a high content of GDF-7 in ligament and tendon tissue and BMP-2 in bone and cartilage (Wolfman et al., 1997). We postulate that either these factors are not present within the tissues or have been not been detected due to our decellularization protocol. Luo et al. (2019) compared various reagents for decellularization of porcine cartilage scaffolds and found the longer the exposure to the decellularizing detergents the less the detected growth factor concentration. The differences of protein distributions in each tissue derived soluble factor were also supported by SDS-PAGE results. The amount of protein factor in fat pad and fat were less than that we expected, which may be caused by less contains of protein in their original tissues or less extraction under the present protocol in such fat-rich tissues and then may result in most of all growth factor could not be detected. Taken together our results suggested that at least 4 kinds of growth factors such as bFGF, IGF-1, VEGF, and TGF-β1 have the tendency of tissue-specific.

To confirm if different tissue-derived soluble ECMs elicited tissue-specific cellular responses, we analyzed their bioactivities by seeding hSMSCs in high density in a 3D collagen gel. As far as the regeneration of musculoskeletal tissues are concerned, SMSCs possess the potential to differentiate into multiple lineages (Fan et al., 2009; Dai et al., 2015; Jacob et al., 2019). Moreover, hSMSCs have already been applied in previous clinical studies in the tissue engineering field (Shimomura et al., 2018). Therefore, hSMSCs were considered to be appropriate to study the differentiation potentials of each soluble ECM. PCR analysis revealed some gene expressions related to each tissue differentiation and maturation showing upregulation in accordance with its origin (Figure 5). SOX9, ACAN, or COL2A1, which are known to be chondrogenic differentiation markers, were upregulated mainly when supplemented with cartilage derived ECM. Addition of meniscus-derived ECM showed upregulation of COL1A1, SOX9, and ACAN, which resembles the meniscal fibrous tissue more than cartilage. SCX, a transcription factor specifically detected in tendon precursor cells (Schweitzer et al., 2001), was likely higher in the groups supplemented with ligament and tendon derived soluble ECMs when compared to their controls or other groups. In the tendon and ligament group, the level of TNC and COL3A1 which are tendon and ECM related genes, were not upregulated when compared to the control. Although the method of cell assay was different from our past study, a similar pattern was found in gene expression profiling in the present study (Yang et al., 2013; Rothrauff et al., 2017a,b; Shimomura et al., 2017). We also noted slight upregulation of desmin in the muscle group. Desmin is an MSC marker, but also expressed in mature myotubes (Kadam et al., 2009). Talovic et al. (2019) reported skeletal muscle derived decellularized ECM gelloids supported MSC differentiation toward myogenic tissue. Their results showed the protein of desmin in MSCs on decellularized ECM gelloids was expressed about three times higher than on pure gelatin gelloids (Talovic et al., 2019). Our results also suggest that muscle-derived soluble ECM has the potential to promote myogenic differentiation to about 1.5 higher than the control although in the gene expression. On the contrary, PPARG, which is transcription factor related with adipogenic differentiation, was not upregulated in fat pad and fat groups. This may result in the ineffective preparation of soluble ECM derived from fat pad or fat tissue. Runx2, which is a master transcription gene for osteoblast differentiation was higher in the cartilage and bone group, although no consequent improvement was demonstrated. However, COL1A1, which is also a marker for bone tissue, was not upregulated significantly but demonstrated expression similar to the control group, while other tissues excluding cartilage and meniscus showed reduced expression. The present cell assay was performed under single 3D condition (such as medium, cell source, tensile loading material of scaffold and cell orientation) to compare the potential of each soluble ECMs purely. More definite differences may be observed by culturing the cells for a longer duration with more appropriate culture conditions and method of decellularization and solubilization for each tissue. Considering the enhanced expression of these markers which are specific to each tissue-derived group, we noted soluble ECMs to be highly bioactive and likely act to promote differentiation toward the native ECM tissue source.

In the present study, soluble factors were extracted using urea. Urea is a chaotropic agent that disrupts hydrogen bonding, resulting in the denaturation of proteins and disruption of lipids and protein interactions (Yang et al., 2013). In a previous study, urea-extracted decellularized ECM had higher concentrations of small and moderate molecular weight proteins compared to pepsin-digested decellularized ECM, which consisted primarily of collagen chains (Yang et al., 2013), therefore we applied a urea extraction protocol to the present study. Guanidine hydrochloride is also known to be effective in extracting heavily cross-linked proteins and proteoglycans from tissues such as tendons or cartilage and applied to other tissues (Vogel and Peters, 2001; Wilson et al., 2010; Barallobre-Barreiro et al., 2017). There is a possibility that a more efficient appropriate decellularization protocol could results in high yield of growth factors with increased bioactivity. To our knowledge, this is the first study to evaluate and compare the biochemical characteristics, growth factor soluble component distribution and bioactivities in nine types of decellularized ECM derived from mesenchymal tissues in the same experiment. In the future such tissue derived soluble ECMs could be employed to regenerate tissues combined with some appropriate scaffolds seeded with some appropriate stem cells. They could be manufactured and delivered as a “bio ink” which would be an efficient natural scaffold solution to print for any defect size and shape matching the recipient site.

Our study is not without limitations in that we didn’t confirm the content of elastin, laminin, lipid, or calcium to prescribe the characteristic of each ECM component. It is unclear how the present protocol could affect them. We only assessed a limited number of growth factors from a large possible number within the tissues. Further variations in growth factors are likely and a more detailed analysis would allow us to draw further conclusions. Finally, we assessed the gene expression profile within 1 week and didn’t confirm the profiling of their synthesized proteins. This should be the next step in our future research to determine the effects of these ECMs on protein synthesis in cultures of longer duration.

## Conclusion

In this study, soluble fractions of nine types of porcine tissues were prepared with a same protocol. Decellularization was successful with reducing cellular component in every tissue and the difference of hydroxyproline and sGAG contain in each native and decellularized tissue was revealed. Moreover, the soluble decellularized ECMs of each tissue exhibited variations in their growth factor distribution and on cell culture appeared to promote cell differentiation toward the specified used ECM tissue phenotype.

## Data Availability Statement

The original contributions presented in the study are included in the article/supplementary material, further inquiries can be directed to the corresponding author.

## Ethics Statement

The studies involving human participants were reviewed and approved by Osaka University Clinical Research Review Committee. Written informed consent to participate in this study was provided by the participants’ legal guardian/next of kin.

## Author Contributions

HH, GJ, and KS contributed conception and design of the study. HH and SN prepared materials. HH and GJ contributed experimental procedures and wrote sections of the manuscript. HH performed the statistical analysis and wrote the first draft of the manuscript. GJ, RT, NN, and KS edited the manuscript. All authors contributed to manuscript revision, read and approved the submitted version.

## Conflict of Interest

The authors declare that the research was conducted in the absence of any commercial or financial relationships that could be construed as a potential conflict of interest.
